# Detection of antibody therapy-induced anti-tumor immune responses using anti-CD8 immuno-pet

**DOI:** 10.1186/2051-1426-3-S2-P391

**Published:** 2015-11-04

**Authors:** Richard Tavare, Helena Escuin-Ordinas, Melissa McCracken, Kirstin Zettlitz, Felix Salazar, Owen Witte, Antoni Ribas, Anna Wu

**Affiliations:** 1 University of California, Los Angeles, Los Angeles, CA, USA; 2 University of California at Los Angeles Medical Center, Los Angeles, CA, USA

## 

Tumor heterogeneity and the dynamic tumor immune microenvironment have become important topics in the field of cancer immunotherapy. The ability to noninvasively monitor immune cells *in vivo* via surface markers on immune cell subsets using immuno-positron emission tomography (immuno-PET) is an attractive means of visualizing both systemic and intratumoral alterations in immune cell numbers and localization during experimental immunotherapies. Due to the critical role of tumor-infiltrating cytotoxic CD8^+^ T cells in anti-tumor immune responses, a radiolabeled anti-CD8 antibody fragment (cys-diabody) was developed for immuno-PET detection of cytotoxic CD8^+^ T cells *in vivo*. The anti-CD8 cys-diabody radiolabeled with ^89^Zr using the bifunctional chelator deferoxamine-maleimide enabled visualization of the spleen and lymph nodes in normal mice; specificity was confirmed in CD8-blocking studies. Next, anti-CD8 immuno-PET was shown to specifically detect tumor-infiltrating lymphocytes in immunotherapy models including agonistic antibody therapy (anti-CD137/4-1BB) and checkpoint blockade antibody therapy (anti-PD-L1). Balb/c mice bearing week old syngeneic CT26 colorectal tumors were treated with either anti-CD137 or anti-PD-L1 antibody therapy and imaged at day 8 post-therapy. All mice treated with anti-CD137 therapy demonstrated tumor regression and increased anti-CD8 immuno-PET targeting in the tumors of mice treated with anti-CD137 therapy compared to the tumor of untreated mice. *Ex vivo* biodistribution confirmed enhanced tumor uptake of anti-CD137 treated versus untreated mice (15 ± 5.5 %ID/g versus 6.0 ± 0.83 %ID/g, respectively). Anti-CD8 immuno-PET of untreated mice demonstrated a rim of activity around the tumor while anti-CD137 treated mice demonstrated enhanced intratumoral uptake. The increase in CD8^+^ tumor-infiltrating lymphocytes was validated by both flow cytometry and immunohistochemistry. About 30% of mice treated with anti-PD-L1 therapy demonstrated tumor regression and were grouped into non-responders (average tumor diameter > 8mm) and responders (average tumor diameter < 8mm). Anti-PD-L1 non-responders and responders had tumor anti-CD8 immuno-PET uptake of 6.4 ± 1.5 %ID/g and 11.27 ± 2.7 %ID/g as determined from *ex vivo* biodistribution analysis, respectively, demonstrating enhanced CD8^+^ T cell infiltration in responding mice that was confirmed by flow cytometry. Anti-CD8 immuno-PET was successful in the noninvasive detection of tumor-infiltrating CD8^+^ T cells in two differing antibody-based immunotherapy strategies that have shown promise in the clinic.

